# Effects of a high-fat meal on inflammatory and endothelial injury biomarkers in accordance with adiposity status: a cross-sectional study

**DOI:** 10.1186/s12937-022-00819-4

**Published:** 2022-10-19

**Authors:** Maria das Graças Coelho de Souza, Priscila Alves Maranhão, Diogo Guarnieri Panazzolo, José Firmino Nogueira Neto, Eliete Bouskela, Luiz Guilherme Kraemer-Aguiar

**Affiliations:** 1 Laboratory for Clinical and Experimental Research on Vascular Biology (BioVasc), Biomedical Center, State University of Rio de Janeiro (UERJ), 20550- 013 Rio de Janeiro, RJ Brazil; 2 Center for Health Technology and Services Research (CINTESIS), Faculty of Medicine, University of Porto, 4200-319 Porto, Portugal; 3 Lipids Laboratory (Lablip), State University of Rio de Janeiro (UERJ), Policlínica Piquet Carneiro, 20550-003 Rio de Janeiro, RJ Brazil; 4 Department of Internal Medicine, Faculty of Medical Sciences, State University of Rio de Janeiro (UERJ), 20551-170 Rio de Janeiro, RJ Brazil; 5 Present Address: Obesity Unit, Centro de Pesquisa Clínica Multiusuário (CePeM), Hospital Universitário Pedro Ernesto (HUPE), State University of Rio de Janeiro, Rio de Janeiro (UERJ), 20551-030 Rio de Janeiro, RJ Brazil

**Keywords:** Obesity, High fat meal, Inflammation, Endothelium, Postprandial period

## Abstract

**Background:**

It is known that consuming a high-fat meal (HFM) induces microvascular dysfunction (MD) in eutrophic women and aggravates it in those with obesity. Our purpose was to investigate if the MD observed after a single HFM intake is caused by endothelial damage or increased inflammatory state, both determined by blood biomarkers.

**Methods:**

Nineteen women with obesity (BMI 30-34.9 kg/m^2^) and 18 eutrophic ones (BMI 20.0-24.9 kg/m^2^) were enrolled into two groups: Obese (OBG) and Control (CG), respectively. Blood samples were collected at five-time points: before (fasting state) and 30, 60, 120, and 180 min after HFM intake to determine levels of adipokines (adiponectin, leptin), non-esterified fatty acid (NEFA), inflammatory [tumor necrosis factor-α (TNF-α), interleukin-6 (IL-6)] and endothelium damage [soluble E-selectin, soluble vascular cell adhesion molecule-1 (sVCAM-1), soluble intercellular adhesion molecule-1 (sICAM-1), plasminogen activator inhibitor-1 (PAI-1)] biomarkers.

**Results:**

Levels of soluble E-selectin, leptin, and PAI-1 were higher in OBG at all-time points (P < 0.05) compared to CG. In the fasting state, OBG had higher levels of NEFA compared to CG (P < 0.05). In intra-group analysis, no significant change in the levels of circulating inflammatory and endothelial injury biomarkers was observed after HFM intake, independently of the group.

**Conclusion:**

Our findings suggest that women with obesity have an increased pro-inflammatory state and more significant endothelial injury compared to eutrophic ones. However, the consumption of a HFM was not sufficient to change circulating levels of inflammatory and endothelial injury biomarkers in either group.

**Registration number for clinical trials:**

NCT01692327.

## Background

Obesity has reached epidemic proportions globally [1]. According to World Health Organization (WHO), in 2016, more than 1.9 billion adults were overweight. Of these, over 650 million had obesity [2], and at least 2.8 million die each year due to obesity [1]. It is well known that unhealthy habits, such as smoking, alcohol abuse and excessive energy drinks consumption, diets with an excess of salt, sugar, high saturated fat, and also discretionary foods, and physical inactivity play a central role in obesity epidemic [3]. All these habits predispose to insulin resistance and dyslipidemia, which increase the risk for obesity-related comorbidities, such as cardiovascular diseases (CVD), type 2 diabetes mellitus (T2DM), nonalcoholic fatty liver disease (NAFLD) and dementia [2, 4, 5]. Nonetheless, CVD increases morbimortality rates being of particular concern [6, 7], in special due to ischemic heart disease and stroke ranking first and second global causes of death, respectively [9].

In addition, recent data have shown that a meaningful hallmark of obesity is chronic low-grade systemic inflammation, an important contributor to atherosclerosis onset [10]. In obesity, visceral adipose tissue downregulates the expression of anti-inflammatory adipocytokines, such as adiponectin, that protects the vascular endothelium and upregulates the expression of several proinflammatory mediators, including C-reactive protein (CRP) and interleukin-6 (IL-6) [11]. Consequently, the endothelium becomes activated and assumes a dysfunctional phenotype [12, 13], expressed by increased leukocyte adhesion, platelet aggregation, hypercoagulation, vascular constriction, and decreased fibrinolysis [14]. Dysfunctional endothelium becomes more permeable and allows low-density lipoprotein (LDL) particles to infiltrate and accumulate in the extracellular matrix, where they undergo oxidation and enzymatic modifications [15, 16]. Modified LDLs exacerbate the proinflammatory process eliciting the secretion of several cytokines, including TNF-α and IL-6, which in turn induce the expression of intercellular adhesion molecule-1 (ICAM-1) and vascular cell adhesion molecule-1 (VCAM-1) on endothelial surface and the release of monocyte chemoattractant protein (MCP-1) among other chemokines, consequently promoting the adherence and migration of monocytes and other immune cells. Once monocytes transmigrate and reach the subendothelial space, they differentiate into macrophages and express high levels of scavenger receptors, allowing them to internalize those modified LDLs [16]. Macrophages then become lipid-laden and convert into foam cells, which is a feature of early fatty streak lesions [17]. In parallel with the first stage of the disease, vascular smooth muscle cells (VSMCs) migrate into the intima, where they deposit extracellular matrix and promote the development of the fibrous cap [15]. As the atherosclerotic plaque progresses, the number of VSMCs decreases and foam cells become apoptotic, releasing metalloproteinases that degrade the fibrous cap, increasing the susceptibility of plaque rupture [18]. The innate and acquired immune responses contribute to the entire atherosclerotic process and are closely related to plaque vulnerability [15]. Thus, endothelial dysfunction (ED) occurs prior to the development of atherosclerotic plaques [14–16] and plays a pivotal role in the progression of CVD [17]. In 1979, Zilversmit proposed that atherosclerosis is a postprandial phenomenon [23] which attracted increased attention to postprandial lipid (PPL) metabolism. High-fat diet alters crucial protective functions of the endothelium, making its phenotype dysfunctional and predisposing it to the development of atherosclerosis [19, 20]. The most striking aspect of ED is the decreased nitric oxide (NO) bioavailability resulting in an impairment of endothelium-dependent vasodilation and loss of its anti-coagulant, anti-adhesive and anti-proliferative properties. The reduced NO bioavailability comes either from reduced production by the endothelium or its enhanced depletion by reactive oxygen species [21, 22].

Several studies have shown the postprandial deleterious effects on vascular function [23, 24]. In addition, our group have demonstrated that a single high-fat meal (HFM) elicited microvascular dysfunction (MD) in eutrophic women and worsened it in those with obesity [25]. However, the underlying mechanisms that induced or aggravated MD after lipid overload remain uncertain but may involve endothelial injury and increased inflammatory status. A study by Nappo et al. 2002 [30] conducted in healthy subjects and in patients with T2DM who ingested a high-fat meal has shown a significant increase in the circulating levels of proinflammatory cytokines, like tumor necrosis factor-α (TNF-α) and IL-6 and of soluble ICAM-1 and VCAM-1, both known as endothelial injury biomarkers. Additionally, Van Oostrom et al. 2003 [31] also studying the effects of high-fat meals demonstrated that they elicited a significant elevation in the number of circulating leukocytes with a concomitant reversible decline of endothelial function.

Based on these findings, this study aimed to investigate if the recorded MD observed after a single HFM intake is caused by endothelial damage or increased inflammatory state both tested by blood biomarkers.

## Methods

### Study Design

This is a cross-sectional study approved by the Research Ethical Committee at Hospital Universitário Pedro Ernesto (CAAE: 0190.0.228.000–10). All participants have read and signed the written informed consent form before being included. This study was registered on the Clinical Trials website under “Study about the HFM and postprandial lipemia” and assigned the identifier NCT01692327.

### Study Population

Patients were recruited in the Obesity Unit for outpatients’ care at the State University of Rio de Janeiro, Brazil. Before inclusion, all participants attended an assessment appointment and underwent clinical, anthropometric, and body composition evaluations and blood collection for biochemical analysis and hemogram.

### Eligibility criteria

The main inclusion criteria were women between 19 and 40 years with body mass index (BMI) between 30 and 34.9 kg/m² (Obesity group - OBG) or between 20 and 24.9 kg/m² (Control group - CG). The exclusion criteria were male sex, hypothyroidism, metabolic syndrome, any degree of glucose intolerance or diabetes mellitus; arterial hypertension; symptoms or history of lactose intolerance; weight gain or reduction by at least 5% of body weight in the past six months before recruitment; tobacco use and alcohol abuse; being involved in a regular practice of physical activity [32] and/or under simultaneous nutritional or pharmacological interventions.

### Blood biochemical analysis

The biochemical analysis encompassed the circulating levels of insulin, total cholesterol (TC), triglycerides (TG), high-density lipoprotein cholesterol (HDL-c), and thyroid-stimulating hormone (TSH) after a 12-hour overnight fast. Fasting and 2-hour post-load (75 g, oral) plasma glucose were also evaluated. Glucose intolerance and metabolic syndrome were defined according to the American Diabetes Association (ADA) criteria[26] and the Joint Interim Statement[34], respectively. Plasma levels of glucose were measured by glucose oxidase colorimetric method. Blood was also collected into serum tubes for insulin and lipid profile analysis. Serum levels of TG, TC and HDL-c were assessed by glycerol phosphate oxidase/peroxidase, cholesterol oxidase/peroxidase and direct colorimetric methods, respectively. All analyses were performed using commercially available kits appropriate for the Automatic Analyser A25 (BioSystems, Barcelona, Spain), following the protocols provided by the kit’s manufacturer (BioSystems, Barcelona, Spain). LDL-c was calculated by the Friedewald equation [26]. Intra and inter-assay coefficient of variation of all analyses described above were below 15% and previously validated [27].

### The test day

**Fig. 1:**
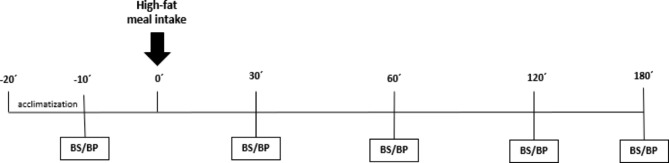
Experimental Design. (BS – blood sample collection; BP – blood pressure)

We included nineteen women in the OBG and eighteen eutrophics who met the eligibility criteria. On the test day, they were asked to arrive after a 12-hour fast at 7:00 a.m. and were placed to rest in an acclimatized room for 20-min. Systolic (SBP) and diastolic (DBP) blood pressures were subsequently assessed by auscultatory method, and then an intravenous catheter was placed and kept in place for blood sample collections during the test. Time points for blood pressure monitoring and blood samples harvesting were: at the fasting state (baseline) and 30, 60, 120, and 180-min after HFM intake (Fig. 1). The same meal was provided to both groups. Participants were instructed not to ingest fat-rich meals, drink caffeine or energy beverages, or practice any physical activity within 24 h before the test.

#### Test meal (high-fat meal)

Participants ingested a single high-fat breakfast consisting of whole milk (200ml), chocolate (10 g), margarine (20 g), croissant (1 unit), cheddar cheese (60 g), and salami (31 g). The meal was similar to the one used by Signori and coworkers[28], with minor changes to incorporate more palatable foods. The meal had 691.5 kcal and consisted of 24.8% carbohydrates, 59.5% lipids (including 21.9 g of saturated fat), and 15.7% proteins. The time limit for its consumption was 10 min.

#### Analysis of inflammatory and endothelial injury biomarkers

Blood samples were collected into EDTA tubes to determine levels of inflammatory [IL-6, tumor necrosis factor-α (TNF-α) and adiponectin] and endothelial injury biomarkers [sE-selectin, plasminogen activator inhibitor-1 (PAI-1), soluble vascular cell adhesion molecule-1 (sVCAM-1) and soluble intercellular adhesion molecule-1 (sICAM-1)] at all-time points. Samples were centrifuged over 10 min at 4^o^C at 1000 g. Plasma was then transferred into cryotubes and stored at -80^o^C until analysis. Plasma levels of sE-selectin, PAI-1, sVCAM-1 and sICAM-1 were assessed by non-magnetic Human Cardiovascular Disease Panel 1 (EMD Millipore Corporation, MA, USA); sensitivity > 0.079 ng/ml. IL-6 was quantified by Quantikine® HS Human IL-6 Immunoassay kit (R&D Systems, MN, USA); sensitivity > 0.039 pg/ml. TNF-α was assayed by High Sensitivity Human Cytokine Magnetic Bead Panel (EMD Millipore Corporation MA, USA); sensitivity > 0.07 pg/ml. Adiponectin was evaluated by Human Adipokine Panel 1 (EMD Millipore Corporation MA, USA); sensitivity > 11 pg/ml. Plasma levels of leptin were determined by Human Metabolic Hormone Magnetic Bead Panel (EMD Millipore Corporation MA, USA); sensitivity > 27 pg/ml. Blood was also collected in serum tubes for non-esterified fatty acids (NEFA) analysis. Serum tubes were centrifuged for 10 min at 18ºC at 3000 rpm. Serum was then transferred into cryotubes and stored at -80ºC until analysis using commercially available reagents (Wako Chemicals, VA, USA). The between and within assay coefficient of variation of all analyses was less than 15%.

### Statistical analysis

Statistical analysis was performed by GraphPad® Prism software, version 5. Normal Gaussian distribution was assessed using the Shapiro-Wilk normality test. For Gaussian and non-Gaussian distributions, data were expressed respectively by mean ± SD or median [1st -3rd quartiles]. Intra-group comparisons were performed by ANOVA repeated measures or the Friedman test. Comparisons between groups were tested by unpaired t-test or Mann-Whitney U test. GPower 3.1.10 software was used for power analysis and sample size estimation. Significant differences were assumed to be present at P < 0.05.

## Results

### Baseline data on body composition, clinical and laboratory evaluation of the participants

Table 1 depicts clinical, laboratory, and body mass characteristics of study participants during the assessment visit. As expected, OBG had significantly greater weight (P < 0.001), BMI (P < 0.001), waist and hip circumferences (P < 0.001), waist-to-hip ratio (WHR, P < 0.001), DBP (P < 0.01), and percent of fat mass (P < 0.001) compared to CG. Additionally, OBG had significantly higher levels of glucose (P < 0.05), insulin (P < 0.01), TC (P < 0.05), LDL-c (P < 0.01), and TG (P < 0.05) than CG. On the other hand, OBG showed significantly lower muscle mass than CG (P < 0.001). There were no significant differences between groups concerning age (P = 0.083), height (P = 0.73), SBP (P = 0.073), heart rate (P = 0.90) and HDL-c (P = 0.33).

**Table 1:** Clinical, laboratory and body composition characteristics of the control group (CG) and group with obesity (OBG).

Variables	CG	OBG
Age (years)	27.89(5.38)	30.74(4.48)
***Anthropometric measures***		
Weight (kg)	57.82(6.24)	86.28(7.84)***
Height (cm)	1.63(0.07)	1.63(0.05)
BMI (kg/m^2^)	21.81(1.79)	32.31(1.54)***
WC (cm)	79.47(5.09)	106.4(9.37)***
HC (cm)	100.3(4.47)	117.5(6.5)***
WHR	0.79(0.03)	0.91(0.85)***
***Blood Pressure and heart rate***		
SBP (mmHg)	109.1(7.53)	115.8(13.82)
DBP (mmHg)	69.09(6.12)	76.74(9.14)**
Heart rate (bpm)	73.19(9.56)	72.84(6.4)
***Body Composition***		
Fat mass (%)	27.88(3.11)	38.12(1.78)***
Muscle mass (%)	72.14(3.08)	61.81(1.75)***
***Laboratorial analysis***		
Glucose (mg/dl)	81.06(6.05)	88.79(13.06)*
Insulin (mU/l)	8.18(4.25)	12.74(4.76)**
TC (mg/dl)	169.40(32.18)	196.50(29.24)*
LDL-c (mg/dl)	86.70(24.92)	110.4(27.81)**
TG (mg/dl)	73.72(37.84)	109.50(56.42)*
HDL-c (mg/dl)	67.94(17.65)	62.37(16.57)

Data are expressed as mean (SD). BMI - body mass index; WC – waist circumference; HP -Hip circumference; WHR - Waist-to-hip ratio; SBP - systolic blood pressure; DBP - diastolic blood pressure; TC- total cholesterol; LDL-c - Low-density lipoprotein cholesterol; TG- triglycerides; HDL-c - High-density lipoprotein cholesterol; *Significantly different compared to CG group; *P < 0.05; **P < 0.01; ***P < 0.001.

### Differences in inflammatory and endothelial injury biomarkers between groups

**Fig. 2:**
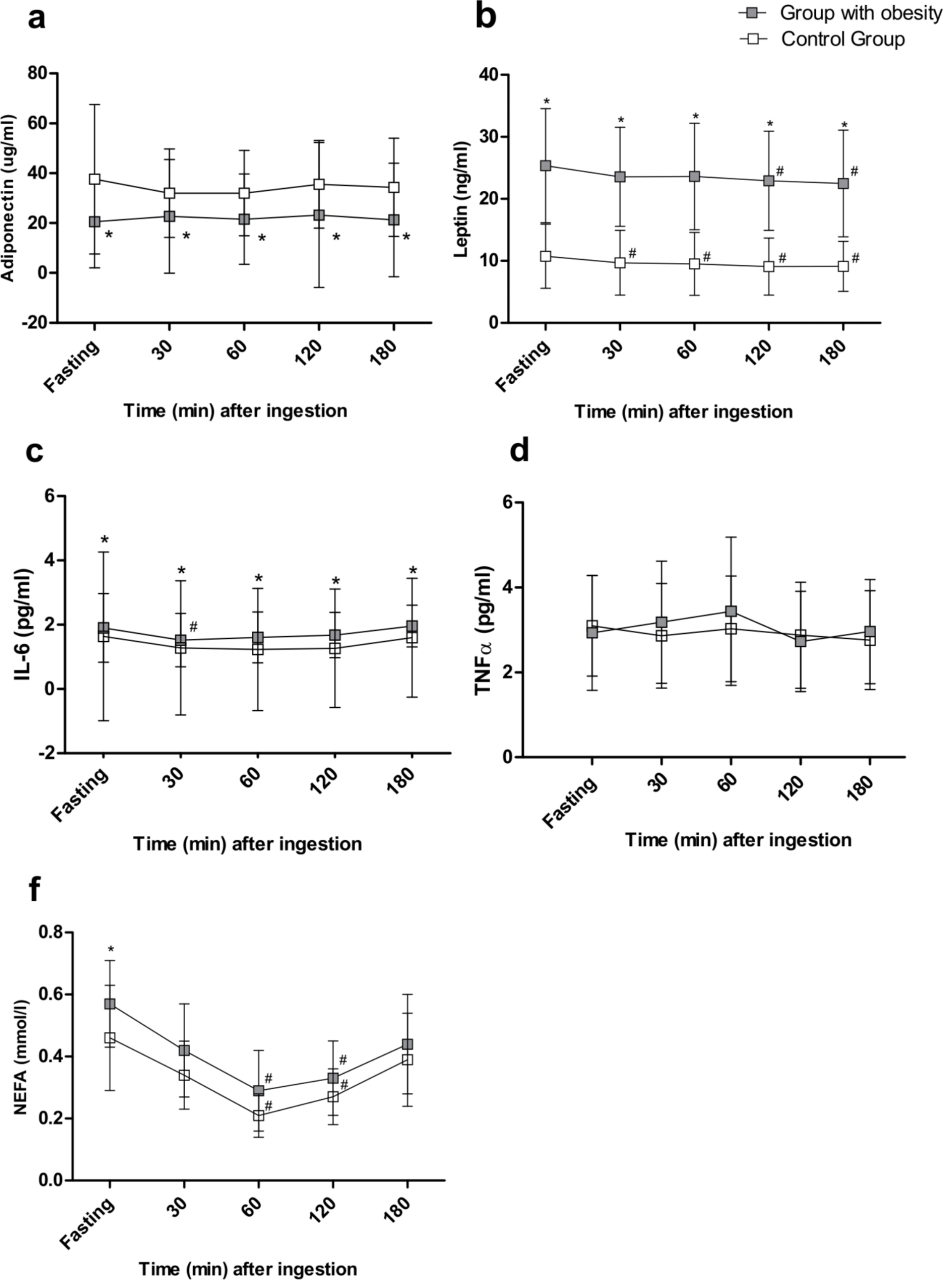
- Adipokines responses elicited by high-fat meal in control (CG) and obese (OBG) groups. (* P < 0.05 intergroup; # P < 0.05 intragroup (compared to baseline))

Regarding adipocytokines (Fig. 2), we did not notice any differences in TNF-α levels between groups in the fasting state. However, at this state, levels of leptin, IL-6 and NEFA were higher in OBG than CG, while adiponectin was lower.

**Fig. 3:**
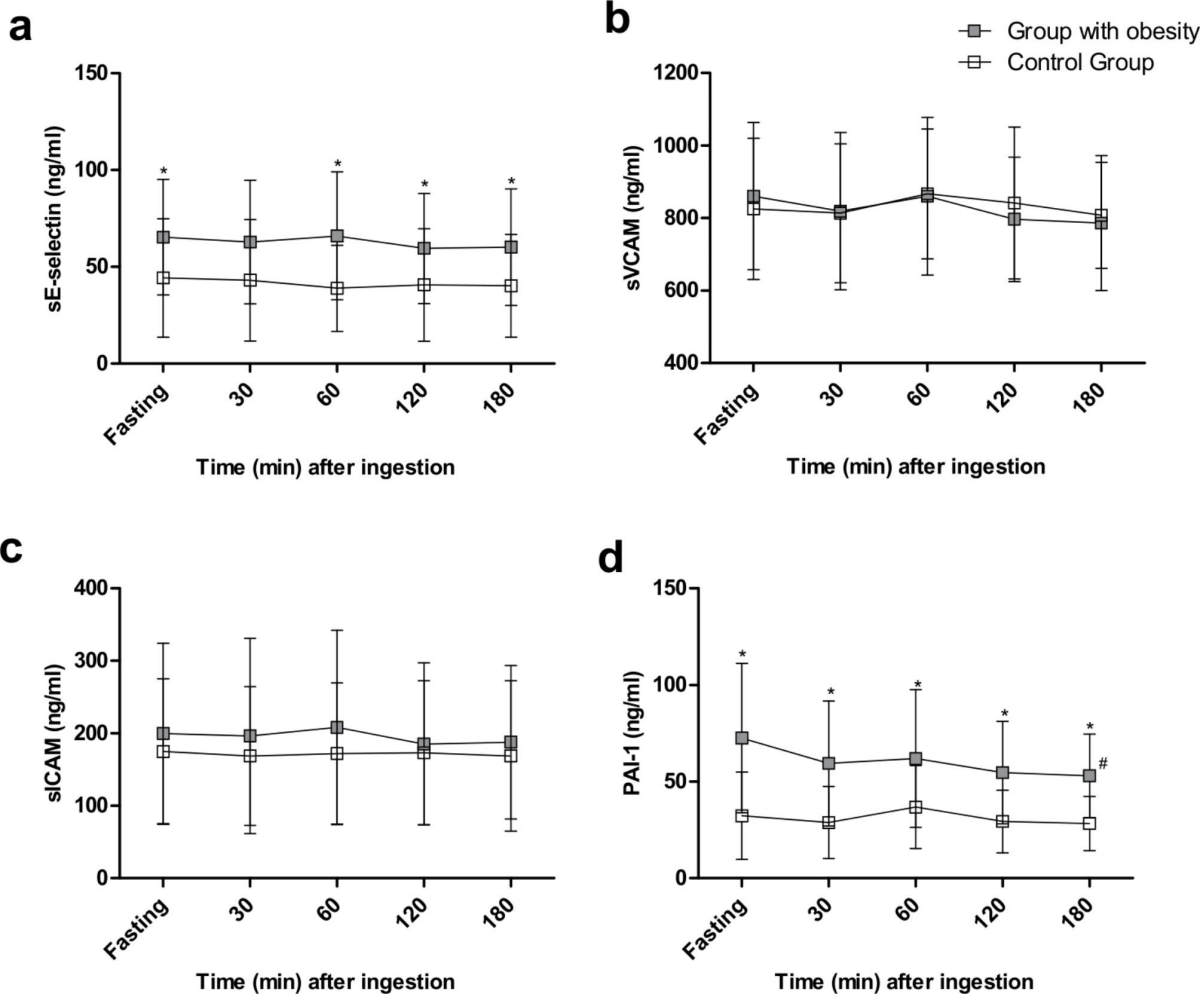
- Endothelial biomarkers responses induced by high-fat meal in control (CG) and obese (OBG) groups. (* P < 0.05 intergroup; # P < 0.05 intragroup (compared to baseline))

Concerning endothelial injury biomarkers, the levels of sICAM-1 and sVCAM-1 were similar between OBG and CG at the fasting state. However, levels of sE-selectin and PAI-1 were higher in OBG than in CG and remained unchanged during the postprandial state (P < 0.05). However, no significant differences were observed in the levels of sICAM-1 and sVCAM-1 during this period (Fig. 3). Regarding adipocytokines, we noticed that leptin and IL-6 levels were significantly higher in OBG than CG during the postprandial period (P < 0.05). In contrast, adiponectin levels were lower (P < 0.05). Furthermore, no significant differences in the levels of TNF-α and NEFA were observed between groups after HFM intake (Fig. 2).

### Differences in inflammatory and endothelial damage biomarkers within groups

No significant differences within groups were noticed regarding endothelial injury biomarkers sE-selectin, s-ICAM-1, and s-VCAM-1 after an HFM intake. However, in OBG, it was observed a significant decrease in PAI-1 levels at 180-min compared to baseline (P < 0.05) (Fig. 3).

Furthermore, NEFA levels at 60 and 120-min after HFM were significantly lower in both groups in comparison to baseline (P < 0.05). The same was observed with respect to leptin levels, that was significantly reduced in both groups after a high-fat meal (OBG at 120-min and 180-min) and (CG at four-time points after HFM ingestion) compared to baseline levels. In addition, we noticed that IL-6 concentration was significantly reduced in OBG (30-min) after meal intake. Nonetheless, no significant differences were observed in adiponectin and TNF-α levels (Fig. 2).

## Discussion

The main finding of this study is that an intake of a single HFM was insufficient to elicit significant changes in inflammatory and endothelial injury biomarkers, independently of BMI. We also observed that women with obesity presented an increased proinflammatory state and endothelium damage before ingesting HFM and sustained it until 180-min after its intake, differently from eutrophic ones. In fact, in the present study, we enrolled participants with obesity who were age and gender-matched to CG. According to exclusion criteria, they were all young, and they did not have T2DM, hypertension or metabolic syndrome, implying that obesity *per se* was responsible for the induction of the proinflammatory state and endothelial injury and not the ingestion of an HFM.

We have reported previously that obesity *per se* elicited MD in women in a fasting state [29]. More recently, we decided to investigate the microvascular function in young women with obesity during the postprandial period [19], considering atherogenesis a postprandial phenomenon. Our findings showed that the metabolic status reflected by changes in glucose, insulin, total cholesterol, HDL, LDL and triglycerides levels after the HFM aggravated MD, which was already present in women with obesity at fasting state and induced the deterioration of microvascular function in eutrophic ones [25]. Nonetheless, the underlying mechanisms responsible for MD after lipid overload remain unclear. We hypothesized that endothelial injury and inflammatory state were probably the main causes of microvascular damage evoked by HFM. In order to answer this question, we examined levels of inflammatory and endothelial injury biomarkers before and after a HFM intake. Adipocytokines, such as leptin and adiponectin, are an essential link between adipose tissue and vasculature, comprising the adipovascular axis, and alterations in their circulating levels will significantly influence vascular function [30]. In addition, we included some inflammatory cytokines, like TNF-α and IL-6, also some endothelial injury biomarkers (such as sE-selectin, sICAM-1, sVCAM-1, and PAI-1), and NEFA because their levels increase in response to subclinical inflammation, playing a significant role in the pathogenesis of the atherosclerotic process [31].

In the present study, we did not observe significant inflammatory and endothelial injury biomarkers changes within groups before and after HFM intake. In contrast, a meta-analysis performed by Tom et al. (2016) revealed that meal consumption reduces flow-mediated dilatation (FMD), evidencing the impairment of endothelial function [39]. The probable explanation for the observed endothelial injury was related to elevation of triglyceride-rich lipids (TRLs) levels after a high-fat consumption, which in turn increased oxidative stress. Oxidative stress is responsible for decreased NO bioavailability reflected by suppressed FMD response, the main feature of ED [40]. Furthermore, humans consume enormous amounts of food generally with high-fat content[34, 35], and consecutive intake of HFM appears to exacerbate lipemia effects[36]. All these factors may contribute to the appearance of atherosclerotic lesions, an independent risk factor for CVD [40].

Interleukin-6 and TNF-α are both critical mediators of inflammation, with increased levels in obesity. Diets may modulate the postprandial response of these two mediators, and we expected that HFM could have a more significant impact on postprandial inflammatory response, particularly in women with obesity. Instead, ingested diet did not change TNF-α levels, while IL-6 levels were reduced at 30-min after HFM. A study by Meyer et al. (2014) corroborates our findings, revealing that after a mixed meal, no significant changes were detected in T2DM and normoglycemic groups 2 and 4-hours after the intake [37]. On the other hand, other studies have reported increased levels of IL-6 in subjects with obesity after HFM ingestion. However, the meals investigated had higher quantities of saturated fat, and levels of circulating IL-6 were assessed for 6 h [19, 38].

Our study also showed that circulating levels of sICAM-1, sVCAM-1, and sE-selectin remained unchanged during the postprandial state in both groups. Corroborating our data, Rubin et al. (2008) did not find any changes in these biomarkers following a lipid-rich test meal. However, these authors pointed out an independent association of postprandial levels of triglycerides with sICAM-1, which could indicate a specific impact of postprandial lipid metabolism on endothelial response [46]. Peairs et al. (2011) claimed that the type of fat in meals could affect postprandial inflammation and endothelial activation differently. In their findings, sICAM-1 levels increased following acute saturated fat ingestion [40]. However, another study found that a meal rich in olive oil elicited a lower postprandial sICAM-1 level than a meal with a higher amount of saturated fat [48].

With regard to adiponectin and leptin, several studies have presented divergent findings [42, 43]. In our study, adiponectin levels in both groups were unaffected by the ingested meal, while leptin levels were reduced in the postprandial state in OBG (from 120-min to 180-min) and CG (from 30-min to 180-min). A recent study found that a fatty meal induces postprandial changes in adiponectin and leptin secretions in normal-weight subjects but not in individuals with obesity, implying that postprandial regulating role of adiponectin and leptin is reduced in obesity [44]. Furthermore, we may speculate that these disparities in postprandial adiponectin and leptin responses are influenced not only by participants’ body weight but also by the meal’s macronutrient composition[45].

Concerning NEFA and PAI-1, no significant changes in their levels following HFM was noticed. However, this is not a consensus since several studies have reported contradicting results. Nonetheless, these studies assessed different populations (e.g. with and without comorbidities), different diets and divergent observation periods following the meals intake [46–50].

Some limitations of our study warrant mention. Ideally, the observation period should be extended from 240 to 360-minutes [51]; however, sequential blood sampling for more than 4 h is very difficult to be performed due to the discomfort inflicted on the participants. Additionally, the literature is controversial with respect to the HFM stimulus. In the present study, it was probably insufficient to cause significant endothelial damage and inflammatory response. We could probably have found different results by adding more saturated fat and testing them for at least three hours or even after three meals over a day. We should have included a regular meal (control meal) to clarify the impact of HFM on inflammatory and endothelial injury biomarkers. We assessed postprandial lipemia only in women. However, Orem et al. (2018) determined postprandial TG ranges in healthy subjects by considering gender differences and noticed that postprandial TG cut-off values for female and male subjects should be determined separately; in other words, postprandial lipemia may display considerable gender differences, and all of them should be studied[52]. Our tests did not consider the women’s menstrual cycle phase. The literature regarding the influence of the menstrual cycle on inflammatory and endothelial biomarkers is controversial. It depends on what biomarker is being tested, adiposity status, the age of the participant, and also on the sample size of the study. Since many disparities occur regarding this issue [60–65], we opted not to fix an exact menstrual cycle phase for the tests, even though this must be viewed as one of our limitations.

## Conclusion

Our findings suggest that women with obesity have an increased proinflammatory state and more significant endothelial damage than eutrophic ones. However, the consumption of a single HFM was insufficient to change the levels of circulating inflammatory and endothelial injury biomarkers. Other factors, like blood viscosity, may be involved in HFM induced microvascular dysfunction. Therefore, more studies are needed to elucidate the underlying mechanisms that induce or aggravate microvascular dysfunction after a single HFM.

## Data Availability

Data will be presented upon forwarding the request to the corresponding author (luiz.aguiar@hupe.uerj.br).

## Abbreviations

ADA: American Diabetes Association
BMI: Body mass index
BP: Blood pressure
BS: Blood sample
CG: Control group
DBP: Diastolic blood pressure
ED: Endothelial dysfunction
HDL: High-density lipoprotein
HFM: High-fat meal
NEFA: Non-esterified free fatty acids
OBG: Obese group
PPL: Postprandial lipemia
SBP: Systolic blood pressure
sICAM: soluble intercellular adhesion molecule-1
sVCAM-1: soluble Vascular cell adhesion molecule − 1
T2DM: Diabetes Mellitus type 2
TRLs: Triglycerides-rich lipoproteins
TSH: Thyroid-stimulating hormone
WHO: World Health Organization
